# Bioactive Glycosaminoglycans from *Caranx crysos*: A Structure–Function Study of Selective Anticoagulant Activity

**DOI:** 10.3390/md24070234

**Published:** 2026-07-03

**Authors:** Ranim Kroumi, Soumaya Alimi, Fabiana Esposito, Asma Haffouz, Basma Hadjkacem, Angela Casillo, Anissa Haddar, Assaad Sila, Emiliano Bedini, Ali Bougatef

**Affiliations:** 1 Laboratory for the Improvement of Plants and Valorization of Agroressources, National School of Engineering of Sfax (ENIS), University of Sfax, Sfax 3038, Tunisia; ranimkroumi99@gmail.com (R.K.); soumayaalimi98@gmail.com (S.A.); anissa.haddar@isbs.usf.tn (A.H.); assaadsila@gmail.com (A.S.); 2 Department of Chemical Sciences, Complesso Universitario Monte S. Angelo, University of Naples Federico II, Via Cintia 4, I-80126 Naples, Italy; fabiana.esposito3@unina.it (F.E.); ebedini@unina.it (E.B.); 3 Laboratory of Molecular Biotechnology of Eucaryotes, Centre of Biotechnology of Sfax, University of Sfax, B.P. 1177, Sfax 3018, Tunisia; haffouzasma@gmail.com (A.H.); basmahadjkacem@gmail.com (B.H.); 4 Department of Life Sciences, Faculty of Sciences of Gafsa, University of Gafsa, Gafsa 2112, Tunisia; 5 Higher Institute of Biotechnology, University of Sfax, Sfax 3000, Tunisia

**Keywords:** *Caranx crysos*, chondroitine sulfate, dermatan sulfate, glycosaminoglycans, chemical characterization, anticoagulant activity, hemolytic activity, cytotoxicity, antiplatelet activity

## Abstract

Glycosaminoglycans (GAGs) are the carbohydrate portion of proteoglycans (PGS), a family of complex biomacromolecules ubiquitously found in the extracellular matrix and on cell surfaces that play critical roles in a plethora of physiological and pathological processes. In the present work, chondroitin sulfate (CS) and dermatan sulfate (DS) were extracted and purified from the head (GCB) and skin (GDB) of blue runner fish (*Caranx crysos*) to explore their structural features and biological properties. GCB and GDB were purified by ion-exchange chromatography with yields of 0.82% and 0.61%, respectively. Chemical and structural analysis showed that GCB and GDD demonstrated quite similar sulfation degrees (4.45% and 4.24%, respectively). The molecular weight values obtained for GCB and GDB as estimated by high-performance size exclusion chromatography coupled with a triple detector array (HP-SEC-TDA) were 48.9 and 28.54 KDa, respectively. Structural features were elucidated using FT-IR and 2D NMR spectroscopy. GCB was mainly identified as chondroitin sulfate, containing 82% GlcA and minor proportions of IdoA and IdoA2S (scoring 18% dermatan-like structures). In contrast, GDB was predominantly dermatan sulfate, with a higher unsulfated IdoA content (54%) and a lower GlcA percentage (17%). In vitro anticoagulant activity, evaluated using APTT and PT assays, demonstrated that both GAGs exhibit significant anticoagulant potential. In addition, both fractions exhibited no antiplatelet activity, suggesting that the isolated glycosaminoglycans selectively target the coagulation cascade without affecting platelet aggregation. Furthermore, hemolytic assays confirmed that neither GCB nor GDB showed any hemolytic activity at the tested concentrations. Cytotoxicity assessment in HEK293 and HUVEK cell lines further confirmed the absence of detectable toxicity even at high concentration. Overall, these marine-derived GAGs present promising therapeutic potential as a source of anticoagulant drugs.

## 1. Introduction

Thromboembolic cardiovascular diseases encompassing deep vein thrombosis, pulmonary embolism and the majority of acute coronary syndromes and ischemic strokes represent a critical global health emergency. Accounting for approximately 18 million deaths annually, i.e., nearly 25% to 30% of global mortality, these conditions remain a leading cause of death worldwide [1,2,3]. Currently, clinical practice uses various classes of anticoagulants for thrombotic disease prevention and treatment. These include heparin (both unfractionated and low molecular weight), vitamin K antagonists like warfarin, direct thrombin inhibitors (argatroban and dabigatran etexilate), and factor Xa inhibitors such as rivaroxaban and betrixaban. Each category targets specific components of the coagulation cascade, offering clinicians therapeutic options tailored to individual patient needs and clinical scenarios [4]. Among these different classes of anticoagulants, heparin occupies a central place in clinical practice due to its effectiveness and historical use in the prevention and treatment of thromboembolic events. Its mechanism of action mainly relies on the potentiation of antithrombin III activity, an intrinsic plasma anticoagulant [5,6]. It can also modulate several biological processes involved in cardiovascular pathophysiology, including inflammatory responses, endothelial function, platelet activation, and vascular remodeling [7]. However, it is important to note that heparin carries the risk of adverse effects including bleeding complications [4,8]. To address these concerns, there is an urgent need to develop novel anticoagulants characterized by an improved safety profile and rapid pharmacodynamic onset. Notably, some sulfated polysaccharides exhibit favorable anticoagulant effects [9,10,11]. Both anticoagulant and antithrombotic activities are associated with the presence of sulfate groups, and their distribution along the polysaccharide chain [12,13]. According to a recent study, new glycosaminoglycans (GAGs) have been isolated from vertebrates such as eels, corbs, flounders, and tunicata as well as marine species like ascidians and sea cucumbers [14,15,16,17,18,19]. The GAGs derived from sea organisms have some advantages over those from domestic sources. In particular, they present a chance to lower the risk of contamination with viruses and prions [20]. Accordingly, the present study investigated the extraction and characterization of biologically active GAGs from fish-derived by-products, thus trying to transform processing waste into high-value resources in a circular bioeconomy frame. In particular, blue runner (*Caranx crysos*) was selected as the target species owing to its recognized importance as a commercially exploited resource in North African and Mediterranean fisheries, particularly in the gulf of Gabes (Tunisia). The substantial volumes of processing by-products are currently discarded with no valorization despite their potential as a source of bioactive compounds [21,22]. To address this, the processing residues were first fractioned into their individual components. GAGs were subsequently extracted through enzymatic digestion, followed by purification using cationic detergent treatment and ethanol precipitation. The identity of the purified glycosaminoglycans was confirmed using a combination of complementary analytical techniques, including sulfate quantification by ion chromatography, Fourier-transform infrared (FT-IR) spectroscopy, HP-SEC coupled with triple detector array (HP-SEC-TDA), and spectroscopy and two-dimensional nuclear magnetic resonance (2D-NMR) spectroscopy, including both homonuclear (COSY, TOCSY) and heteronuclar (^1^H,^13^C-DEPT-HSQC) experiments. Together, these analyses verified that the isolated fractions corresponded to chondroitin sulfate/dermatan sulfate glycosaminoglycans with the expected structural features. In addition, the purified GAGs were evaluated for their anticoagulant activity.

## 2. Results and Discussion

### 2.1. Extraction, Purification and Chemical Composition Analysis

**Figure 1:**
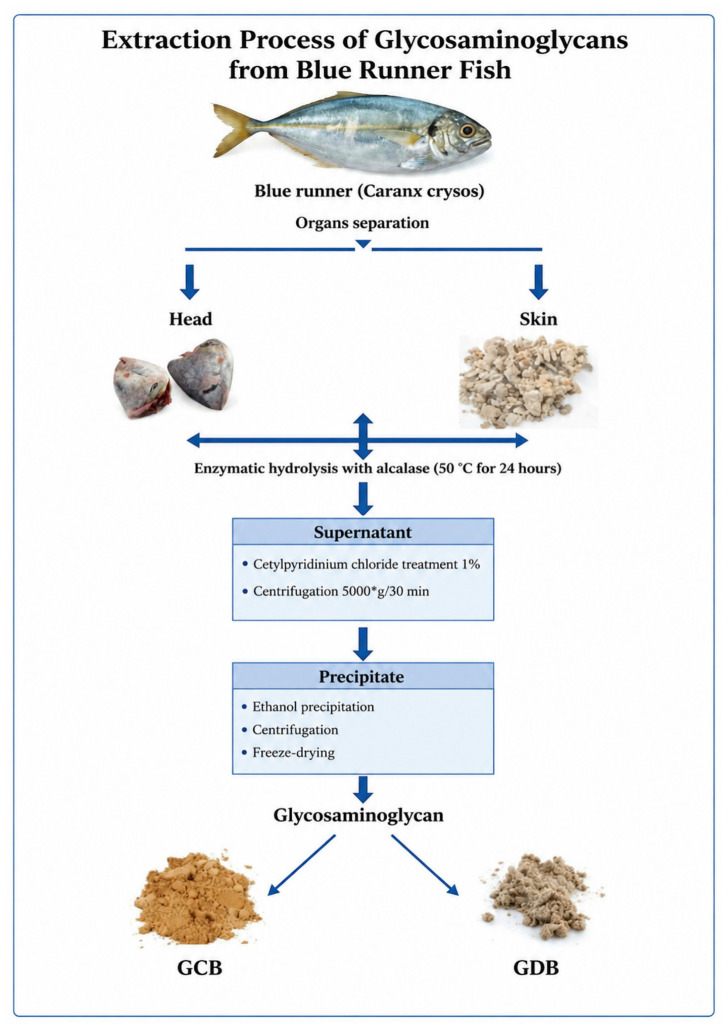
Extraction procedure of GAGs isolated from blue runner head (GCB) and skin (GDB).

GAGs are complex carbohydrate-based biomacromolecules ubiquitously distributed throughout the animal kingdom. Through their interactions with a wide range of proteins they are involved in physiological and pathological processes. GAGs mediate diverse biological processes including tissue repair and wound healing [23], modulation of coagulation and thrombotic pathways [24], regulation of tumor development [25], and mediation of inflammatory responses [26], which underlie their extensive use in medicine as therapeutic agents [27,28]. In this study, GAGs were extracted from the head (GCB) and skin (GDB) of the blue runner (*Caranx crysos*) through enzymatic hydrolysis by digestion with Alcalase^®^, followed by separation using DEAE-cellulose column chromatography (Figure 1).

The physicochemical properties and extraction yield of GAGs isolated from the two fish tissues are summarized in Table 1. Based on dry weight, the GAG yield was most prominent in the head tissues (0.82%) and skin (0.61%). For comparison, the yields of isolated GAGs from the head and skin of garfish (*Belone belone*) were around 8.1% and 3.7%, respectively [29]. The sulfate content of GCB and GDB was determined by ion chromatography (IC) and gave similar values (4.45 ± 0.01% and 4.24 ± 0.08%, respectively). The measured sulfate contents were lower than those previously reported for glycosaminoglycans extracted from Sciaena umbra (28.74%) [16] and Nephrops norvegicus 23% [30]. These findings suggest that the origin, the nature of the raw material, and the extraction method significantly influence the sulfate content. The color values for GCB and GDB are also presented in Table 1. The former exhibited lower lightness (L* = 59.48) but higher redness (a* = 9.73) compared to the latter (L* = 67.66; a* = 5.26), while yellowness (b*) values were similar (GCB: 26.29; GDB: 25.47). GDB L* value was similar to that previously described [31] for the glycosaminoglycans extracted from gray triggerfish and smooth hound (L* = 66.395% and 68.26%, respectively).

### 2.2. FT-IR Spectroscopy

**Figure 2:**
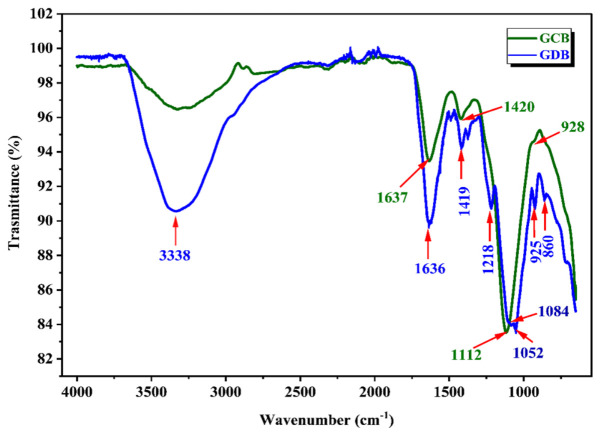
FT-IR spectra of GCB and GDB.

Fourier-transform infrared (FT-IR) spectroscopy was first used to elucidate the structural characteristics of GCB and GDB, with a focus on identifying specific organic functional groups associated with their polysaccharide backbones. As shown in Figure 2, the bands at 3338 cm^−1^ represented the stretching of the hydroxyl groups. The bands observed at around 1636 cm^−1^ were attributed to the stretching vibrations of C=O bonds of the acetamido group [32]. The peaks near 1419 cm^−1^ were assigned to the carboxylate groups COO- [33]. It is noteworthy that the bands observed at 1218, 1112, 1084, 1052 and 925 cm^−1^ were characteristic of the presence of sulfate groups [34].

### 2.3. Molecular Weight Analysis

Molecular weight analysis of GAGs is crucial for understanding their structure, function, and quality, particularly in pharmaceutical and biomedical fields. High-performance size exclusion chromatography (HP-SEC), combined with advanced detectors, is widely used to determine the molecular weight distribution of polymers and proteins. The results of two analyses for GCB and GDB in terms of M_w_ (weight average molecular weight), M_n_ (number average molecular weight), polydispersity index (expressed as M_w_/M_n_ ratio), and intrinsic viscosity (IV_w_ (dL/g)) are reported in Table 2; GCB exhibited a higher molecular weight, with a Mw value of approximately 48.9 kDa, whereas GDB showed a lower value for both M_w_ (28.54 kDa) and dispersity (1.286).

### 2.4. Structural Analysis by NMR Spectroscopy

**Figure 3:**
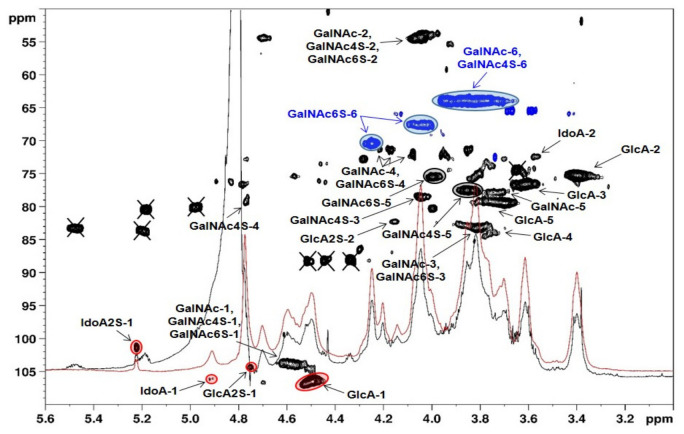
Zoomed superimposition of 1H- (black), 1D-DOSY (red) NMR and 1H,13C-DEPT-HSQC 2D-NMR (black: CH and CH3; blue: CH2) (600 MHz, 298K, D2O) of GCB with assignment of the most characteristic cross-peaks. Signals enclosed in blue and black were relatively integrated for DSGalNAc-6 and DSGalNAc-4 estimation, respectively, while signals enclosed in red circles were relatively integrated for DSGlcA-2, DSIdoA-2 and chondroitin-/dermatan-type subunits ratio estimation. Crossed signals in the 1H,13C-DEPT-HSQC spectra are related to impurities, as determined by the comparison of 1H- and 1D-DOSY spectra.

**Figure 4:**
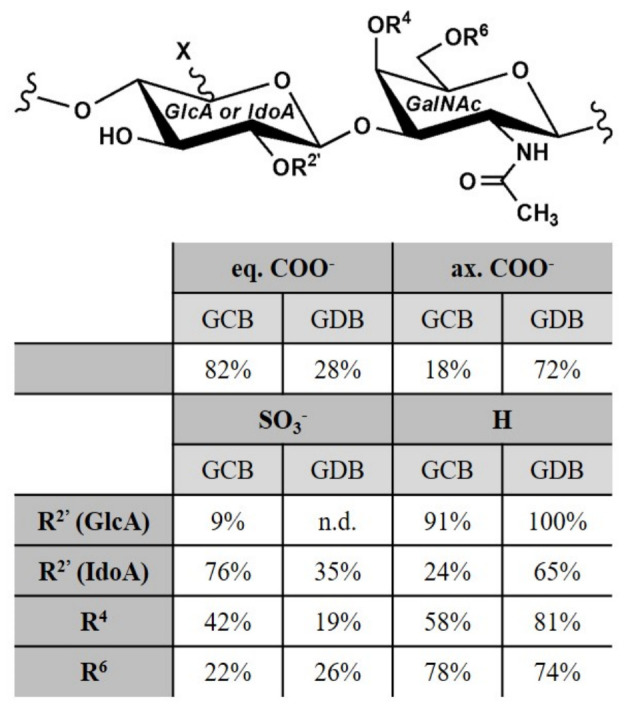
Structural features of GCB and GDB chondroitin/dermatan hybrid polysaccharides (eq. = equatorial; ax. = axial; n.d. = not detected).

**Figure 5:**
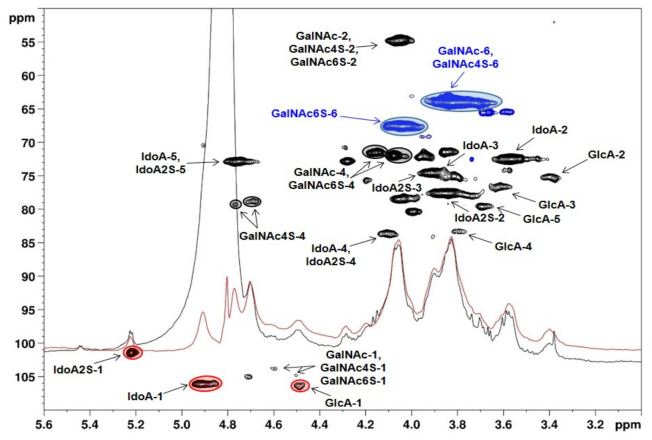
Zoomed superimposition of 1H- (black), 1D-DOSY (red) NMR and 1H,13C-DEPT-HSQC 2D-NMR (black: CH and CH3; blue: CH2) (400 MHz, 298K, D2O) of GDB with assignment of the most characteristic cross-peaks. Signals enclosed in blue and black were relatively integrated for DSGalNAc-6 and DSGalNAc-4 estimation, respectively, while signals enclosed in red circles were relatively integrated for DSGlcA-2, DSIdoA-2 and chondroitin-/dermatan-type subunits ratio estimation.

The fine structural features of GCB and GDB were investigated by a set of homonuclear and heteronuclear two-dimensional NMR spectra (COSY, TOCSY and ^1^H,^13^C-DEPT-HSQC; see Figure 3, Figure 4 and Figure 5 and Figures S1 and S2). The ^1^H,^13^C-DEPT-HSQC spectrum of GCB (Figure 3) revealed the presence in the anomeric region of two major cross-peaks centered at δ_H,C_ 4.58/103.4 and 4.49/106.4 ppm that could be assigned to β-linked 2-acetamido-2-deoxy-D-galactose (*N*-acetyl-galactosamine, GalNAc) and D-glucuronic acid (GlcA) units, respectively, by comparison with literature [35]. The former peak was also confirmed by the detection of a correlation with the signal at 4.04 ppm in the COSY spectrum, which corresponds to a cross-peak in the ^1^H,^13^C-DEPT-HSQC spectrum at δ_C_ 54.3 ppm, a chemical shift value characteristic for a CH-2 of aminosugars, while the latter was confirmed by COSY correlation with H-2 signal at 3.40 ppm and TOCSY correlations with H-3 and H-4 signals at 3.62 and 3.71 ppm, that are all typical chemical shift values for a β-gluco-configured residue. Minor anomeric cross-peaks were detected at δ_H,C_ 5.22/101.3 and 4.92/106.2 ppm, and assigned to differently sulfated α-linked L-iduronic acid (IdoA) [36], i.e., the C-5 epimer of GlcA with an axially—rather than equatorially—oriented COO-. This suggested that GCB consisted of a major amount of chondroitin-type subunits together with a minor quantity of dermatan disaccharide residues, in agreement with reports indicating that some natural chondroitin sulfates contain limited epimerization domains, resulting from partial epimerization of GlcA to IdoA [37]. The detection of hybrid chondroitin/dermatan polysaccharide chains is not unprecedented in marine organisms [38].

In order to investigate the sulfation pattern on GCB, assignment of ^1^H,^13^C-DEPT-HSQC cross-peaks related also to CH_2_ and non-anomeric CH atoms was done, through a combined analysis of homonuclear (COSY and TOCSY) and heteronuclear 2D-NMR (^1^H,^13^C-DEPT-HSQC) spectra as well as a comparison of the measured ^1^H and ^13^C chemical shifts with literature data [35,36,39,40,41]. As expected, chondroitin-type subunits were found to be sulfated highly predominantly on GalNAc residues, either at primary hydroxyl on position 6 or at secondary hydroxyl at position 4. Unsulfated GalNAc units were also detected. By assuming that signals associated with the same CH or CH_2_ atoms in sulfated and unsulfated units display similar ^1^J_C_,_H_ coupling constants and that a difference of approximately 5–8 Hz from the experimental set value does not cause a substantial variation in the integrated peak volumes [42,43], the degree of sulfation at GalNAc-4 and -6 position (DS_GalNAc-4_ and DS_GalNAc-6_, respectively) could be estimated. To this aim, signals related to CH-5 of 4-*O*-sulfated (GalNAc4S), 6-*O*-sulfated (GalNAc6S) and unsulfated GalNAc units could have been selected for the relative integration, because they were clearly distinguished from each other in the ^1^H,^13^C-DEPT-HSQC spectrum (δ_H/C_ 4.00/75.4, 3.85/77.5 and 3.74/77.6 ppm for GalNAc6S-, GalNAc4S- and GalNAc-CH-5, respectively). Unfortunately, the signal at δH/C 3.74/77.6 ppm was partially overlapped with the cross-peak assigned to GlcA CH-5 atoms. However, DSGalNAc-6 could be estimated to be equal to 0.22 by relative integration of the cross-peaks at δH/C 4.25/70.3, 4.00–4.10/67.1 and 3.70–3.94/63.9 ppm. Indeed, the first two signals could be assigned to CH_2_-6 atoms of GalNAc6S units involved in chondroitin- or dermatan-type subunits, respectively, while the latter cross-peak was attributed to CH_2_-6 atoms of GalNAc and GalNAc4S residues. Moreover, the relative integration of the well-resolved GalNAc6S- and GalNAc4S-CH-5 signals afforded a 6-*O*-/4-*O*-sulfation ratio equal to 0.53, thus allowing an estimation of DS_GalNAc-4_ value (0.42). To complete the description of the structural features of GCB by NMR, a relative integration of the anomeric signals δ_H/C_ at 5.22/101.4, 4.92/106.1, 4.75/104.3 and 4.49/106.4 ppm—assigned to IdoA2S, IdoA, GlcA2S and GlcA, respectively—allowed to estimate the chondroitin-/dermatan-type subunits ratio (82:18) and the degree of sulfate decoration at 2-O-site of both GlcA (DS_GlcA-2_ equal to 0.09) and IdoA units (DS_IdoA-2_ equal to 0.76). A summary of the structural features of GCB chondroitin/dermatan hybrid polysaccharide is depicted in Figure 4.

In contrast to GCB, the anomeric region of the ^1^H,^13^C-DEPT-HSQC 2D-NMR spectrum of GDB (Figure 5) showed a clear predominance of IdoA residues, indicating a dermatan sulfate-rich composition. The chondroitin-/dermatan-type subunits ratio could be estimated to be equal to 28:72 by relative integration of the anomeric cross-peaks related to GlcA, GlcA2S, IdoA and IdoA2S, as already described for GCB. This result is in agreement with the known, pivotal role for dermatan sulfate in shaping the structural and functional integrity of skin [44] through the higher flexibility of IdoA ring with respect to GlcA [45]. The estimation of DS_GalNAc-6_, DS_GalNAc-4_, DS_GlcA-2_ and DS_IdoA-2_ was also done, as already discussed above for GCB. All DS values of GDB were lower with respect to GCB, but DS_GalNAc-6_, for which a slight increase was estimated (Figure 4). It is worth noting that a single signal related to CH_2_-6 atoms of GalNAc6S units could be found in the spectrum, while GCB showed two of them, as discussed above. In particular, only the cross-peak at δ_H/C_ 4.00–4.10/67.1 ppm—assigned to GalNAc6S residues involved in dermatan-type subunits—could be found in the ^1^H,^13^C-DEPT-HSQC 2D-NMR spectrum of GDB, thus revealing that sulfation at position 6 occurred exclusively on dermatan domains of GDB. Indeed, sulfation at the C6 position of GalNAc in dermatan sulfate is known to contribute mainly to its structural diversity and to influence its interactions with biologically active proteins [46,47].

### 2.5. In Vitro Anticoagulant Activity

The clinical utility of standard thrombin and factor Xa inhibitors is frequently compromised by adverse bleeding events. This therapeutic limitation has driven a dedicated search for novel anticoagulants with improved safety profiles [48,49]. It is now well-established that certain polysaccharides can exert biological effects, particularly GAGs, which are widely recognized for their anticoagulant properties. Research has demonstrated that these polysaccharides exhibit anticoagulant activity through multiple mechanisms along the coagulation cascade. Their inhibitory effects include blocking the initial activation of the extrinsic pathway as well as suppressing tenase and thrombin activity [50,51]. In this context, to provide a comprehensive pharmacological profile of GCB and GDB, their biological evaluation was designed as a multi-assay strategy covering both efficacy and safety aspects. The aPTT and PT assays were performed as complementary tests to assess the anticoagulant activity through the intrinsic and extrinsic coagulation pathways, respectively, while cytotoxicity, hemolytic, and antiplatelet activity assays were included to evaluate their biocompatibility and complete their antithrombotic profile. Notably, in the study of anticoagulant mechanisms, aPTT (activated partial thromboplastin time) and PT (prothrombin time) are widely used as standard coagulation assays [52]. aPTT measures the functionality of the intrinsic pathway by assessing coagulation factors VIII, IX, and XI, while PT evaluates the extrinsic and common pathways via factors I (fibrinogen), II (prothrombin), V, VII, and X [53,54].

**Figure 6:**
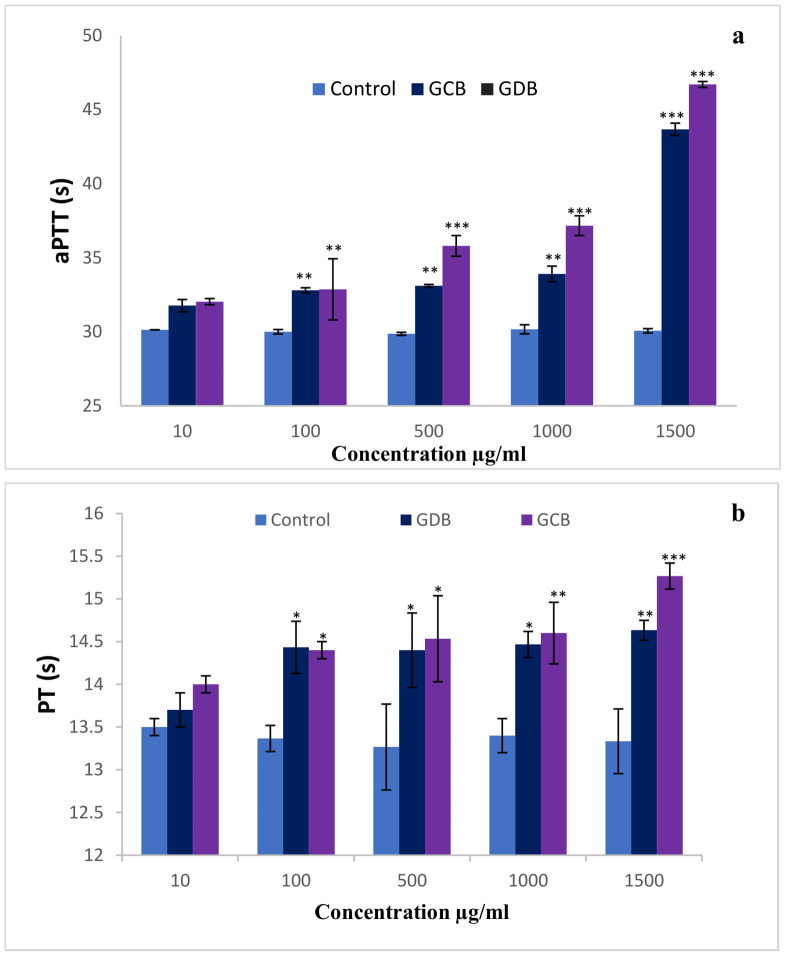
Anticoagulant activity of GCB (head) and GDB (skin) at different concentrations evaluated by the measurement of (**a**) activated partial thromboplastin and (**b**) prothrombin time. Plasma samples were preincubated with the respective GAGs for 3 min at 37 °C prior to coagulation assays. Values are expressed as mean ± SEM (*n* = 3). Statistical significance was determined by Student’s *t*-test compared to the negative control group. * *p* < 0.05; ** *p* < 0.01; *** *p* < 0.001.

The anticoagulant activity of GCB and GDB determined by the aPTT test is shown in Figure 6a. Briefly, plasma samples were preincubated with the respective GAGs for 3 min at 37 °C prior to coagulation assays. The results showed that both GAGs induced a strong and progressive dose-dependent prolongation of aPTT across all tested concentrations (10–1500 µg/mL) compared to their respective negative controls (pooled plasma without GAGs) measured at each concentration point. The negative control values remained stable throughout the experiment, ranging from approximately 30.0 s to 30.1 s, confirming the consistency and reliability of the assay conditions. At the highest tested concentration of 1500 µg/mL, GCB and GDB induced significant prolongations of clotting time, reaching 43.6 s and 46.7 s, respectively, compared to a control value of 30.0 s. Notably, GDB demonstrated superior anticoagulant potency relative to GCB. This observed prolongation of aPTT suggests that these GAGs primarily inhibit the intrinsic and/or common coagulation pathways. In a similar context glycosaminoglycans extracted from the heads and skin of garfish (*Belone belone*) respectively prolonged the aPTT at a concentration of 1000 µg/mL to values about 2.93 and 4.05 times greater than that of the negative control [29]. To further characterize the anticoagulant properties of GCB and GDB, the PT assay was performed to assess their influence on the extrinsic coagulation pathway (Figure 6b). The negative control values, consisting of pooled plasma without GAGs, remained remarkably stable across all tested concentrations, ranging from 13.0 s to 13.5 s. In contrast to the aPTT results, both GAGs elicited a modest prolongation of PT, which became detectable primarily at the highest concentrations. At the maximum tested concentration of 1500 µg/mL, GCB and GDB extended the clotting time to 14.6 s and 15.2 s, respectively, representing a slight but significant increase relative to the negative control value of 13.3 s. These results demonstrate that while these GAGs have a pronounced effect on the extrinsic pathway (up to 50%), they exhibit a lower inhibitory effect on the PT test, which is at best around 15% at high concentrations. In the same context, sulfated glycosaminoglycans from the skin of European eel significantly prolonged PT specifically (at a concentration of 500 µg/mL, the PT was 1.24-fold higher than that of the control) [14]. The observed disparity in potency between the aPTT and PT assays highlights a distinct pathway selectivity for GCB and GDB, which is closely associated with their structural parameters. It is well-established that the anticoagulant activity of sulfated polysaccharides is largely mediated by their interaction with antithrombin (AT), which subsequently inhibits serine proteases in the coagulation cascade, particularly factors IXa, Xa, XIa, and thrombin (IIa) [55,56]. This mechanism predominantly affects the intrinsic and common pathways, explaining the strong aPTT prolongation observed here. Conversely, the extrinsic pathway (initiated by the Factor VIIa-Tissue Factor complex) is generally less sensitive to AT-mediated inhibition by GAGs, accounting for the modest PT prolongation [57]. This selectivity is governed by specific structural features, including the degree of sulfation (DS), molecular weight (Mw), sulfate group positioning, and monosaccharide composition. Higher DS values and increased molecular weights are generally associated with enhanced binding affinity to antithrombin and greater anticoagulant activity [55]. Therefore, the results suggest that GCB and GDB recovered from the head and skin of blue runner possess structural motifs optimized for modulating intrinsic blood coagulation factors and thrombin activity, rather than the extrinsic pathway. This profile is advantageous as it suggests potential anticoagulant efficacy with a potentially lower risk of bleeding complications associated with extrinsic pathway inhibition.

### 2.6. Hemolytic Activity

The hemolytic potential of GAGs extracted from the head (GCB) and skin (GDB) of blue runner (*Caranx crysos*) was evaluated using human erythrocytes. Across a broad concentration range (10 to 50 mg/mL), neither GCB nor GDB induced significant hemolysis. These results demonstrate that both extracts are non-hemolytic, suggesting a favorable safety profile and a lack of cellular toxicity even at high concentrations.

### 2.7. Effect of the Isolated Glycosaminoglycans on Platelet Aggregation

The isolated glycosaminoglycans were evaluated for their effects on platelet aggregation, the first stage of hemostasis that precedes activation of the coagulation cascade. They were evaluated at 1 mg/mL, corresponding to the concentration showing anticoagulant activity in this study, to assess their effect on platelet aggregation under identical experimental conditions, enabling a direct comparison of both activities. Platelets were treated with the glycosaminoglycans (1 mg/mL) or with distilled water (0.5%) as a vehicle control, then stimulated with ADP (20 µM). As shown in Table 3, neither the GCB nor the GDB inhibited platelet aggregation at the tested concentration, with inhibition levels not exceeding 11% for any sample. In contrast, the reference inhibitor indomethacin (0.5 mM) markedly suppressed ADP-induced aggregation.

These results suggest that the isolated GCB and GDB selectively target the coagulation pathway without affecting platelet aggregation. Such specificity is advantageous, as simultaneous inhibition of both pathways is known to markedly increase the risk of bleeding [56].

### 2.8. Toxicity Assessment of the Isolated Glycosaminoglycans

**Figure 7:**
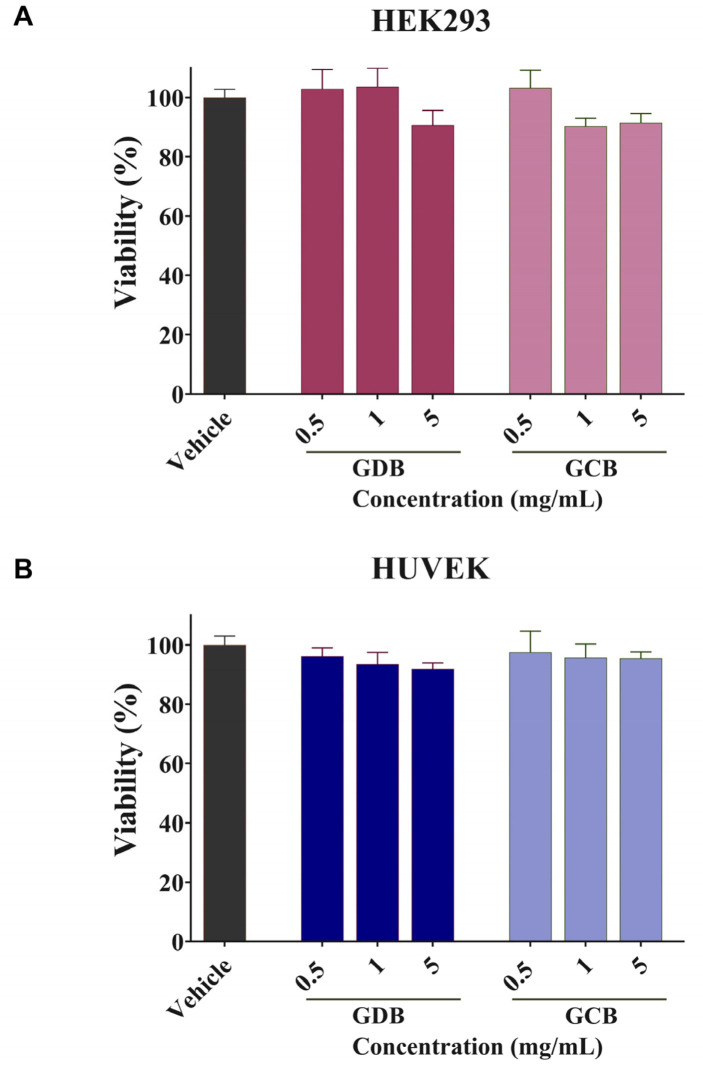
Toxicity assessment of the isolated glycosaminoglycans. (**A**) Cell viability of HEK293 cells and (**B**) cell viability of HUVEK cells, assessed after 24 h of treatment with the tested glycosaminoglycans (0.5–5 mg/mL). Data are expressed as mean ± SD of three independent experiments. Distilled water was used as a vehicle control for the dissolution of the samples.

The toxicity of the tested GCB and GDB samples was evaluated on Human Embryonic Kidney 293 (HEK293) cells and Human Umbilical Vein Endothelial (HUVEK) cells in view of their potential therapeutic applications. HEK293 cells were selected as a representative model of human non-cancerous renal cells, widely used to assess general systemic cytotoxicity and potential nephrotoxicity [57], while HUVEC cells were chosen as a well-established in vitro model of vascular endothelial function, relevant to cardiovascular and antithrombotic studies [58]. The results demonstrated that the glycosaminoglycans were well tolerated, showing no significant cytotoxicity in either cell line at concentrations up to 5 mg/mL, with cell viability remaining above 90% in all tested conditions (Figure 7A) and (Figure 7B) compared with the distilled water-treated control. This favorable safety profile supports their suitability for further preclinical assessment. The absence of toxicity on HEK293 cells suggests a low risk of renal toxicity, which is particularly important considering the kidney’s central role in drug elimination [59]. Likewise, the absence of cytotoxicity in HUVEC cells indicates good compatibility with vascular endothelial cells, an essential requirement for compounds intended for cardiovascular applications. Overall, these findings provide strong preliminary evidence of GCB and GDB polysaccharides’ biocompatibility and their potential as promising anticoagulant candidates.

## 3. Materials and Methods

### 3.1. Reagents

The analytical-grade chemical substances and solvents used in this study were obtained from commercial suppliers, including Sigma-Aldrich (St. Louis, MO, USA). The Bacillus licheniformis protease (Alcalase^®^ 2.4L) was kindly provided by Novozymes^®^ (Copenhagen, Denmark).

### 3.2. Preparation of Materials

Fresh blue runner (*Caranx crysos*) fishes were freshly purchased from the local fish market in Sfax, Tunisia. After being sealed in polyethylene bags and kept on ice, the biological materials were delivered to the research laboratory in less than 30 min. After arriving, the samples (skin and head) were cleaned twice with water in order to remove impurities. Before being used for the extraction and analysis of GAGs, the samples were collected and kept at −20 °C in sealed plastics bags.

### 3.3. Extraction of Glycosaminoglycans from Blue Runner via Enzymatic Hydrolysis

GAGs were extracted following a known protocol [31]. In brief, 5 g of powder blue runner (*Caranx crysos*) waste (head and skin) was mixed separately with 250 mL sodium acetate buffer (0.1 M, pH 8.0). Enzymatic hydrolysis was then conducted using Alcalase^®^ (Novozymes^®^, Copenhagen, Denmark) at 50 °C for 24 h. Following hydrolysis, the mixture was incubated at 80 °C for 20 min to denature endogenous enzymes, then cooled to ambient temperature prior to filtration. The remaining solid residue was washed with distilled water and re-filtered. The pooled filtrates were treated with 1% (*w*/*v*) cetylpyridinium chloride (CPC) to precipitate the GAGs. After a 24 h incubation at room temperature, the resulting solids were recovered by centrifugation at 5000× *g* for 30 min at 4 °C. The pellets were then dissolved in a minimal volume of aq. 2M NaCl ethanol mixture (100:15, *v*/*v*) and incubated at 4 °C for 24 h. Precipitation of GAGs was carried out twice with absolute ethanol, once each for 24 h, at 4 °C and subsequently by centrifugation at 5000× *g* for 30 min. The final GAG pellet was subsequently dissolved in deionized water and lyophilized for storage.

### 3.4. Purification of GAGs from Blue Runner Skin and Heads

The purification of the extracted GAGs was achieved employing diethylaminoethyl-cellulose (DEAE-cellulose) ion-exchange chromatography. GAGs were initially dissolved in distilled water, then subjected to a DEAE-cellulose (2 × 6 cm) column that was previously washed with a 50 mM NaCl solution. The components retained on the column were eluted sequentially with 50 mM NaCl and 2.5 M NaCl, then precipitated with three volumes of ethanol at −20 °C for 24 h. After centrifugation (7500× *g*, 15 min) the pellet was lyophilized and stored at −20 °C for future analysis.

### 3.5. Determination of GAGs Yield

The yields of the two glycosaminoglycans extracted from the skin and heads of blue runner were calculated using the following equation:

Yield (%) = (weight of dried GAGs (g)/weight of dry raw material (g)) × 100

### 3.6. Determination of Sulfate Content

Sulfate determination was performed using IC on a Thermo Scientific Dionex Aquion system equipped with an IonPac AS14A anion-exchange column (Thermo Scientific, Waltham, MA, USA). A 10 μL aliquot of either the sample or standard solution was directly injected into the chromatographic system. The mobile phase, composed of 8 mM sodium carbonate and 1 mM sodium bicarbonate, was delivered at a flow rate of 1.0 mL/min. The total analysis time was 15 min.

### 3.7. Determination of Color

The color characteristics of the GAGs were evaluated using a CHROMA METER CR-400/410 (Konica Minolta, Chiyoda, Japan), operating under the CIE Lab color system (C/2°). Each sample was positioned between two steel dishes featuring a 5.7 cm diameter circular opening to ensure consistent measurement conditions. The color parameters are defined as follows: L* represents lightness (ranging from 0 = black to 100 = white), a* indicates chromaticity on the red-green axis (positive values for red, negative for green), and b* denotes chromaticity on the yellow-blue axis (positive values for yellow, negative for blue). A standard white reference tile (L* = 93.68, a* = −0.69, b* = −0.88) was used to calibrate the device. Measurements were taken at ambient temperature at five different locations on each film, and the reported results correspond to the average of these five readings.

### 3.8. Infra-Red Spectroscopic Analysis

FT-IR absorption spectra were recorded in the 450–4000 cm^−1^ range using a Nicolet spectrometer (Thermo Fisher Scientific, Waltham, MA, USA). The measurements were performed on KBr pellets containing 0.1% of the samples to obtain transmission spectra.

### 3.9. Molecular Weight Determination

Molecular weight analysis was performed using Gel Permeation Chromatography (GPC)/Size Exclusion Chromatography (SEC) GPC/SEC system combined with integrated multi-detectors: refractive index (RI), UV/Vis absorbance (UV) and light scattering (LS). Samples (1 mg/mL) were dissolved in 0.1 M aqueous NaNO_3_ solution and filtered using disposable 0.22 μm syringe filters. Chromatographic analyses were performed under isocratic conditions, by using aqueous NaNO_3_ solution. The prepared samples were then examined using a Malvern Panalytical A6000M columns (Malvern Panalytical, Enigma, UK) at a flow rate of 0.8 mL/minute with injection volumes of 100 µL.

M_w_, M_n_, polydispersity index, and intrinsic viscosity IV_w_ were calculated by the sample concentration and according to the following formulae associated with the signals of the three detectors, as previously reported [60,61]:

RI = K_1_·dn/dc·c;

UV = K_2_·IV_w_·c;

LS = K_3_·M_w_·(dn/dc)^2^·c

where c is the sample concentration (mg/mL), dn/dc is the refractive index increment, and K_1_, K_2_ and K_3_ are optical instrumental constants that includes the wavelength and refractive index effects. By solving the equations, the system allowed the simultaneous determination of c, M_w_, and IV_w_. Universal calibration for the determination of K_1_, K_2_ and K_3_ was performed by using a polyethylene oxide (PEO) standard (22 kDa PolyCAL, Viscotek). The dn/dc used for the calculation was 0.148 mL/g, as already reported for other GAGs [62].

### 3.10. NMR Spectroscopy Acquisition and Parameters 

NMR spectra were acquired using either an Avance NEO spectrometer (^1^H: 600 MHz, ^13^C: 150 MHz) equipped with a cryogenic probe or an Avance III HD spectrometer (^1^H: 400 MHz, ^13^C: 100 MHz) from Bruker (Billerica, MA, USA). All samples were dissolved in D_2_O, and acetone was used as internal standard (^1^H: δ = 2.22 ppm; ^13^C: δ = 31.5 ppm). Spectra were analyzed with TopSpin software (version 4.0.5, Bruker). Gradient-selected COSY and TOCSY spectra were recorded with spectral widths of 6000 Hz in both dimensions with data sets of 2048 × 300 points; TOCSY experiments were performed using a mixing time of 120 ms. Two-dimensional ^1^H,^13^C-DEPT-HSQC spectra were recorded in ^1^H-detected mode on single quantum coherence, with proton decoupling in the ^13^C dimension, using data sets of 2048 × 300 points and typically 100 increments.

### 3.11. In Vitro Anticoagulant Activity Assays

Blood samples were collected from healthy volunteers who had abstained from taking any medication, particularly aspirin or other anti-inflammatory drugs, for at least 15 days. Written informed consent was obtained from all donors. Blood was drawn into trisodium citrate (3.2% *w*/*v*; 1:9 dilution). To obtain platelet-rich plasma (PRP), samples were centrifuged at 1000 rpm for 10 min. The remaining blood was then centrifuged at 3500 rpm for 5 min to prepare platelet-poor plasma (PPP). Serum was prepared by allowing blood to clot at room temperature followed by centrifugation. These preparations were used in subsequent anticoagulant and antiplatelet assays.

The influence of GAGs on hemostatic regulation was assessed by measuring the activated partial thromboplastin time (aPTT) and prothrombin time (PT) using a semi-automated coagulometer KC4 Delta, the samples were dissolved in physiological serum, all analyses were conducted in triplicate and mean values were taken.

#### 3.11.1. Activated Partial Thromboplastin Time Assay

The activated partial thromboplastin time (aPTT) was measured by incubating 45 µL of normal citrated platelet-poor plasma (PPP) with 5 µL of purified GAGs at various concentrations for 3 min at 37 °C. Subsequently, 50 µL of aPTT reagent (CK-PREST^®^) was added followed by incubation for another 3 min at 37 °C. Clot formation was initiated by adding of 100 µL of 25 mM CaCl_2_ and the clotting time was measured. Results were expressed in seconds and as a ratio, with a normal reference value below 1.2. For the enzyme activity control (C), physiological serum replaced GAG samples.

#### 3.11.2. Prothrombin Time Assay

The prothrombin time (PT) was assessed by pre-incubating 45 μL of PPP with 5 µL of GAGs at various concentrations (37 °C for 3 min), Coagulation was initiated by adding 100 μL of Neoplastine^®^ CI (Diagnostica Stago Milano, Milan, Italy). The PT value was expressed in seconds.

### 3.12. Hemolytic Activity Assays

The hemolytic activity of GCB and GDB was evaluated according to the method described by [57] using human blood samples provided by the Chaker university hospital (Sfax, Tunisia). The results obtained after treating erythrocytes with only NaCl/Pi buffer and SDS (0.2%) were taken as negative control (0% lysis of erythrocytes) and positive control (100% lysis of erythrocytes), respectively.

### 3.13. Antiplatelet Activity

The antiaggregant activity of GCB and GDB was evaluated using the microplate aggregometry method developed by [63] with minor modifications [64,65]. Both compounds (1 mg/mL) were incubated with PRP for 30 min at 37 °C. Distilled water (0.5%) served as the negative control, and indomethacin was used as the reference antiplatelet drug. Afterwards, 140 µL of PRP or GCB and GDB-treated PRP was transferred to microplate wells containing 20 µM ADP (Sigma-Aldrich Merck, Darmstadt, Germany). Absorbance was recorded at 630 nm every 20 s with shaking at 1020 rpm using a Varioskan Flash plate reader (Thermo Scientific, Waltham, MA, USA). All experiments were performed at least in triplicate.

### 3.14. Cytotoxicity Assay

The cytotoxicity of the tested GCB and GDB was evaluated on HEK293 and HUVEK cell lines (obtained from the American type culture collection (ATCC)) using the MTT (3-(4,5-dimethylthiazol-2-yl)-2,5-diphenyltetrazolium bromide) assay. Cells were maintained in Dulbecco’s Modified Eagle Medium (DMEM) supplemented with 10% fetal bovine serum, 1% L-glutamine, and 50 μg/mL gentamicin at 37 °C in a humidified 5% CO_2_ atmosphere until confluence. Cells were seeded at a density of 10^4^ cells/mL in 96-well plates and allowed to adhere overnight. They were then treated with GAG samples at different concentrations (0.5–5 mg/mL) or distilled water (vehicle) and incubated for 24 h. All experiments were conducted in triplicate. Subsequently, MTT solution (5 mg/mL) was added to each well and incubated for 4 h. After removing the supernatant, formazan crystals were dissolved in DMSO, and absorbance was measured at 570 nm using a Varioskan Flash plate reader (Thermo Fisher Scientific, Waltham, MA, USA) [66].

### 3.15. Statistical Analysis

Data are expressed as means ± standard deviation (SD). Statistical analysis was performed with SPSS-22 software (SPSS-IBM, Armonk, NY, USA) by one-way ANOVA followed by Tukey’s test. *p*-values < 0.05 (*) are considered statistically significant, <0.01 (**) very significant, and <0.001 (***) highly significant.

## 4. Conclusions

In summary, GAGs were successfully extracted from by-products of blue runner fish (*Caranx crysos*) processing, with GCB obtained from the head and GDB from the skin. These GAGs were structurally characterized using FT-IR and by two-dimensional NMR spectroscopy. The results revealed that both GCB and GDB possessed hybrid chondroitin/dermatan sulfate polysaccharide structure, with a marked predominance of chondroitin subunits in GCB (82%) and of dermatan subunits (72%) in GDB. Sulfate group decorations occurred mainly on GalNAc residues, as typical for chondroitin- and dermatan-type GAGs, with a predominance at *O*-4 site in GCB and at *O*-6 position in GDB. For both samples, additional sulfate groups could be detected also on uronic acid units, exclusively at *O*-2 position. Molecular weight determination by HP-SEC-TDA revealed values of 48.9 kDa for GCB and 28.54 kDa for GDB. Both GAGs exhibited potent anticoagulant activity, as evidenced by significant prolongation of aPTT and PT values, while showing no effect on platelet aggregation. Interestingly, no cytotoxicity was observed in erythrocytes, HEK293, or HUVEC cells even at high concentrations.

These findings suggest that fish-derived chondroitin sulfate and dermatan sulfate could serve as promising bioactive compounds in pharmaceutical and biomedical contexts, offering sustainable alternatives to mammalian sources.

## Figures and Tables

**Table 1:** Physicochemical properties and extraction yields (calculated based on dry matter) of GDB and GCB.

**Yield**	**GCB**	0.82%
	**GDB**	0.61%
**Sulfate**	**GCB**	4.45 ± 0.05%
	**GDB**	4.24 ± 0.08%
**Color**	**GCB**	L* 67.66 a* 5.26 b* 25.47
	**GDB**	L* 59.48 a* 9.73 b* 26.29

**Table 2:** HP-SEC-TDA data for GCB and GDB.

	GCB	GDB
Mn (g/mol)	31.939	22.129
Mw (g/mol)	48.927	28.545
Mw/Mn	1.532	1.286
IVw (dl/g)	0.5506	0.3968

**Table 3:** Effect of the isolated glycosaminoglycans on ADP-induced platelet aggregation. Results are expressed as mean ± SD of three independent experiments (*n* = 3).

Sample	Distilled Water (0.5%)	Indomethacin (0.5 mM)	Glycosaminoglycans (1 mg/mL)	
			GDB	GCB
Inhibition (%)	2.5 ± 1.7	44.7 ± 6.2 ***	11.0 ± 3.5	7.8 ± 2.5

*** *p*-value < 0.001: The results were significantly different compared to the distilled water-treated control platelets.

## Data Availability

The data presented in this study are available on request from the corresponding author.

## Supplementary Materials

The following supporting information can be downloaded at: https://www.mdpi.com/article/10.3390/md24070234/s1. Figure S1: Zoomed superimposition of 1H-NMR and COSY (black) and TOCSY (red) 2D-NMR spectra (600 MHz, 298K, D2O) of GCB. Figure S2: Zoomed superimposition of 1H-NMR and COSY (black) and TOCSY (red) 2D-NMR spectra (400 MHz, 298K, D2O) of GDB.
