# Supplementary material for: Bioactive Glycosaminoglycans from Caranx crysos: A Structure–Function Study of Selective Anticoagulant Activity
Source: Mar Drugs. 2026 Jul 3;24(7):234. doi: 10.3390/md24070234 (PMC13412997; doi:10.3390/md24070234)
Supplement: Supplementary file 1 [file marinedrugs-24-00234-s001.zip › marinedrugs-4328103-supplementary.pdf]

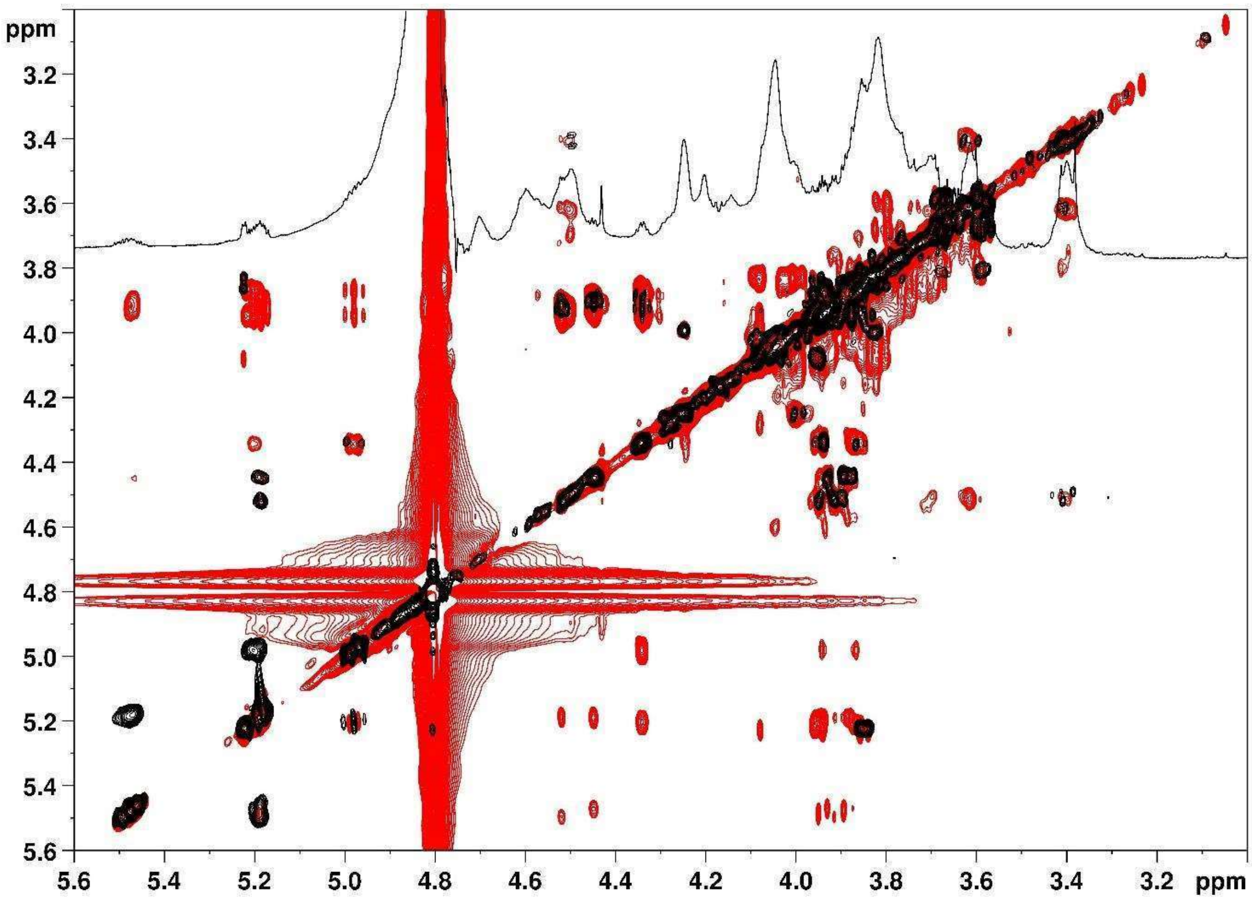

Figure S1: Zoomed superimposition of ^{1}H-NMR and COSY (black) and TOCSY (red) 2D-NMR spectra (600 MHz, 298K, D_2O) of GCB

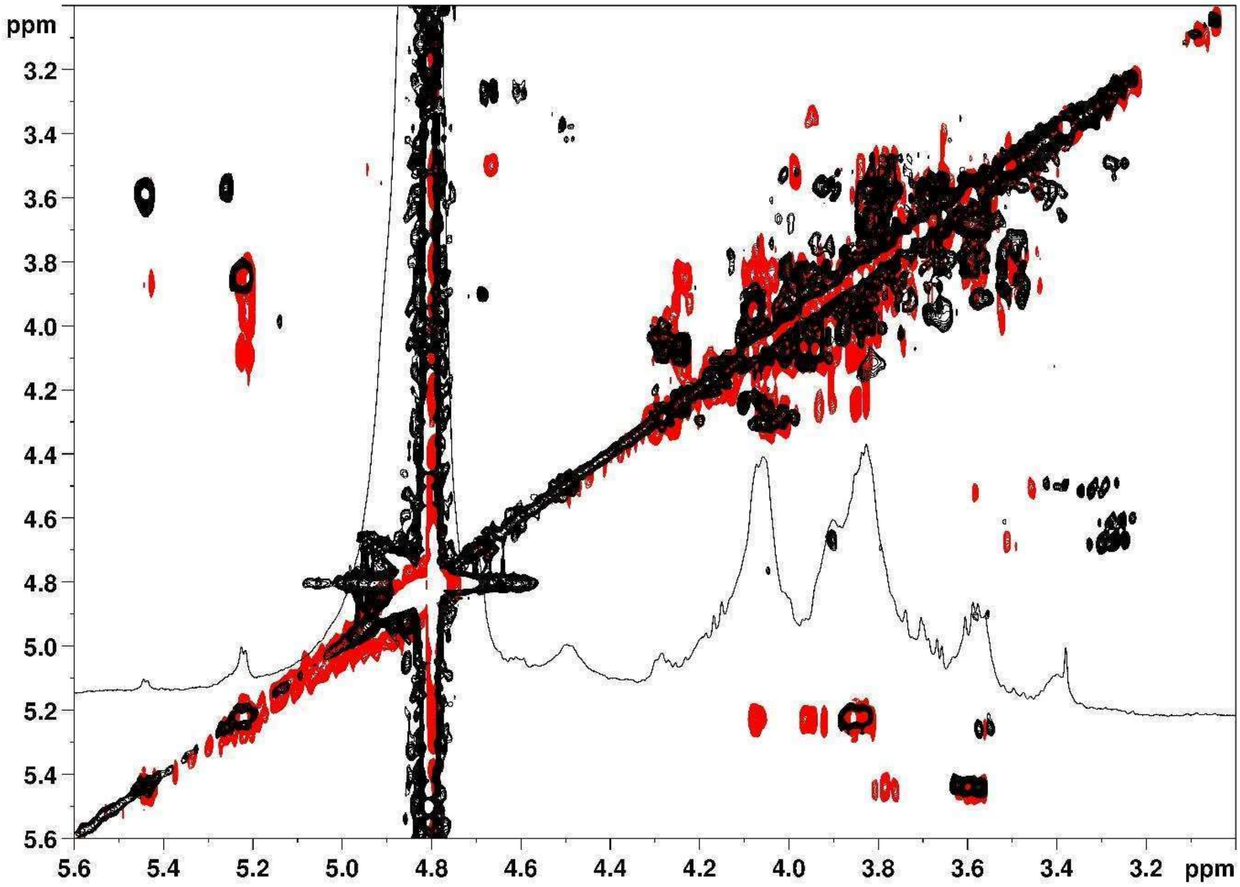

Figure S2: Zoomed superimposition of ^{1}H-NMR and COSY (black) and TOCSY (red) 2D-NMR spectra (400 MHz, 298K, D_2O) of GDB
